# Fetal Congenital Heart Disease Caused by Compound Heterozygous Mutations in the *DNAH9* Gene: A Case Report

**DOI:** 10.3389/fgene.2021.771756

**Published:** 2022-01-18

**Authors:** Tao Zhang, Hua Yuan, Hongdan Zhu, Yuyi Ying, Jinlong Ding, Haigang Ding, Xiaoliang Shi, Yao He, Haitao Pan, Yongxing Zhong

**Affiliations:** ^1^ Shaoxing Maternity and Child Health Care Hospital, Shaoxing, China; ^2^ Obstetrics and Gynecology Hospital of Shaoxing University, Shaoxing, China

**Keywords:** congenital heart disease, DNAH9 gene, copy number variation sequencing, whole exome sequencing, 3D structure

## Abstract

**Background:** Fetal congenital heart disease (CHD) is the most common congenital defect, with an incidence of 0.6–0.8%, accounting for 30–50% of infant congenital disease deaths. The pathogenesis of CHD is still unclear, so an active and effective prenatal diagnosis is very important for the prevention and control of CHD. Herein, a Chinese CHD patient with rare compound heterozygous mutations in the *DNAH9* gene was reported, and the 3D structure and functional changes of *DNAH9* protein were predicted.

**Case presentation:** A 23-year-old pregnant woman came to our hospital for prenatal diagnosis at 27 weeks of gestation. Both she and her partner were unaffected. Fetal CHD was detected by ultrasound screening. Copy number variation sequencing (CNV-seq) revealed an 81 kb deletion at chr17p12 (11,486,795–11,568,385), including exons 1–15 of *DNAH9* gene, which plays a key role in cardiac development. Then, whole exome sequencing (WES) was used and identified a nonsense mutation (c.10975C>T) in *DNAH9*, which resulted in the mutation of amino acid 3,659 from glutamine to termination. The 3D mutant protein structures were predicted using SWISS-MODEL and showed structural changes from functional β-sheet and α-helix to termination, respectively.

**Conclusion:** We describe a case of fetal CHD caused by *DNAH9* mutations and provide an effective diagnostic technique for identifying intragenic deletions. This diagnostic process can be implicated in prenatal diagnosis of CHD.

## Introduction

Congenital heart diseases (CHD) are the most common birth defects, which can severely affect human health, accounting for 2–8% of newborn children (Hoffman et al., 2004; Lambrechts et al., 2005). The clinical manifestations and severity of CHD are widely variable. Mild cases, such as some small ventricular septal defects, can self-close after birth. Severe cases, such as most instances of tetralogy of Fallot (TOF), seriously affect the function and structure of the heart, with a high mortality. CHD is often accompanied by multiple organ malformations, which is one of the main causes of neonatal death (Pediatric Cardiac Genomics Consortium et al., 2013; Egbe et al., 2014). Traditional thought is that CHD is the combined result of environmental and genetic factors, in which environmental factors are dominant (Kalisch-Smith et al., 2020). However, with the development of technologies, it has been found that the incidence of CHD is significantly higher in monozygotic twins, or when patients have family history of CHD or consanguinity. A variety of chromosomal aberrations is often associated with different types of CHD. It is now highly suggested that genetic factors play an important role in the pathogenesis of CHD (Van der Bom et al., 2011). In particular, the invention and application of chromosome microarray technology (CMA) and high-throughput sequencing technology provide a strong basis for elucidating the extremely important role of genetic factors in the occurrence of CHD (Charron et al., 2010; Rehm, 2013; Aburawi et al., 2015).

Monogenic inherited disease refers to disease caused by a single gene abnormality, also known as Mendelian genetic disease. Monogenic disease can be divided into autosomal dominant or recessive, X-chromosome dominant or recessive genetic diseases. The incidence rate of any individual monogenic disease is not high, but the overall incidence rate of all monogenic diseases combined is 4–5%. This kind of genetic disease can also be seen in various CHD, which can be an isolated CHD phenotype (such as in *GATA4* and *NKX2.5* gene mutations (Winston et al., 2012)) or a part of complex syndrome, such as Noonan syndrome or Holt-Oram syndrome.

*DNAH9* gene associated primary ciliary dyskinesia 40 is characterized by chronic respiratory tract infection, visceral translocation and infertility (Fassad et al., 2018; Loges et al., 2018). Most of the patients with primary ciliary dyskinesia have congenital respiratory diseases. From early childhood, patients will have repeated respiratory infections. However, the respiratory compromise of primary ciliary dyskinesia type 40 is relatively light, and usually does not develop into serious lung disease. About 50% of patients with primary ciliary dyskinesia have mirror image inversion of internal organs, and some patients have been reported to have severe congenital heart malformations.

In this study, we reported the case of a Chinese fetal proband, who presented with fetal CHD by ultrasound screening. Copy number variation sequencing (CNV-seq) revealed an 81 kb deletion at chr17p12, including a deletion of exons 1–15 in *DNAH9*. Then, whole exome sequencing (WES) revealed a nonsense mutation (c.10975C>T) in *DNAH9* on the other allele. This base mutation resulted in the codon of amino acid 3,659 from glutamine to termination. To predict the changes of 3D protein structure, we used SWISS-MODEL and PyMO and revealed these two mutations changed functional β-sheet and α-helix structures, respectively.

## Materials and Methods

### Sample Collection

The study was approved by the institutional ethics committee of Shaoxing Maternity and Child Health Care Hospital. The family members had signed informed consent documents. Parental consent was obtained for collecting the prenatal fetal cord venous blood at 27 weeks of pregnancy. Peripheral blood samples also were collected from proband’s parents.

### Copy-Number Variation Sequencing and Whole Exome Sequencing

Genomic DNA was extracted from the proband and parental blood using a DNEasy Blood and Tissue Kit (Qiagen, Hilden, Germany) according to the manufacturer’s procedures, respectively. For WES, the genomic DNA of the proband was enriched for coding exons using Agilent SureSelect Low Input Reagent Kit and sequenced on Illumina HiSeq X Ten platform. The sequencing data captured 99.75% of coding regions across 35,519,957 bp length of 25,701 genes in total. The average sequence depth is 180.347X and 97.87% of targeted regions with average depth >20X. For CNV-seq, the genomic DNA was fragmented using Hieff NGS^®^ Fast-Pace^TM^ DNA Fragmentation Reagent and prepared for the PCR-free library by Hieff NGS^®^ Complete Adapter Kit for Illumina^®^.

### Data Analysis

The AfterQC (Chen et al., 2017) was used to evaluate the sequencing quality of the original sequencing data, and the low quality and contaminated reads were removed. After data were aligned to human reference hg19 by BWA software (Li and Durbin, 2010), the single nucleotide variants (SNV) and indels in genome were called by using the GATK software (Mckenna et al., 2010). Then, we used 1000 Genomes database (1000 human genome dataset), Genome AD (Genome Aggregation Database dataset) 2.1.1, and ExAC (The Exome Aggregation Consortium dataset) to screen the SNV and indels and the OMIM, HGMD, and Clinvar databases to filter the reported mutations. dbNSFP database was used to predict the pathogenicity of missense mutation and splice mutation. All mutation sites were classified by ACMG genetic variation classification criteria and guidelines. Finally, Sanger sequencing method was used to verify all possible pathogenic sites.

### Protein Structure Prediction

The protein sequence with 4,486 amino acid residues of *DNAH9* was download from NCBI (NP_001363.2). The wild-type and mutant-type 3D structure of the *DNAH9* protein was predicted using SWISS-MODEL web server (https://swissmodel.expasy.org/) (Waterhouse et al., 2018). The best model was selected based on QMEANDisCo global score. The final predicted structure was visualized using PyMOL program (https://pymol.org/).

## Results

### Clinical Features

At 31 weeks of gestation, the parents were fully informed of severe fetal deformity and the significance of the compound *DNAH9* mutations. The parents strongly requested the induction of labor. With the approvement of the institutional ethics committee of Shaoxing Maternity and Child Health Care Hospital, the pregnancy was terminated for fetal anomalies. The parents and other families were in normal physical condition, without any family history of genetic diseases. Prior to the proband, her mother had two histories of adverse pregnancies: 1) At 26 weeks of gestation, pregnancy was terminated due to the discovery of fetal single chamber heart and 2) at the third month of another pregnancy, spontaneous abortion occurred. Her mother’s menstruation was irregular in the period of 30–40 days.

**FIGURE 1:**
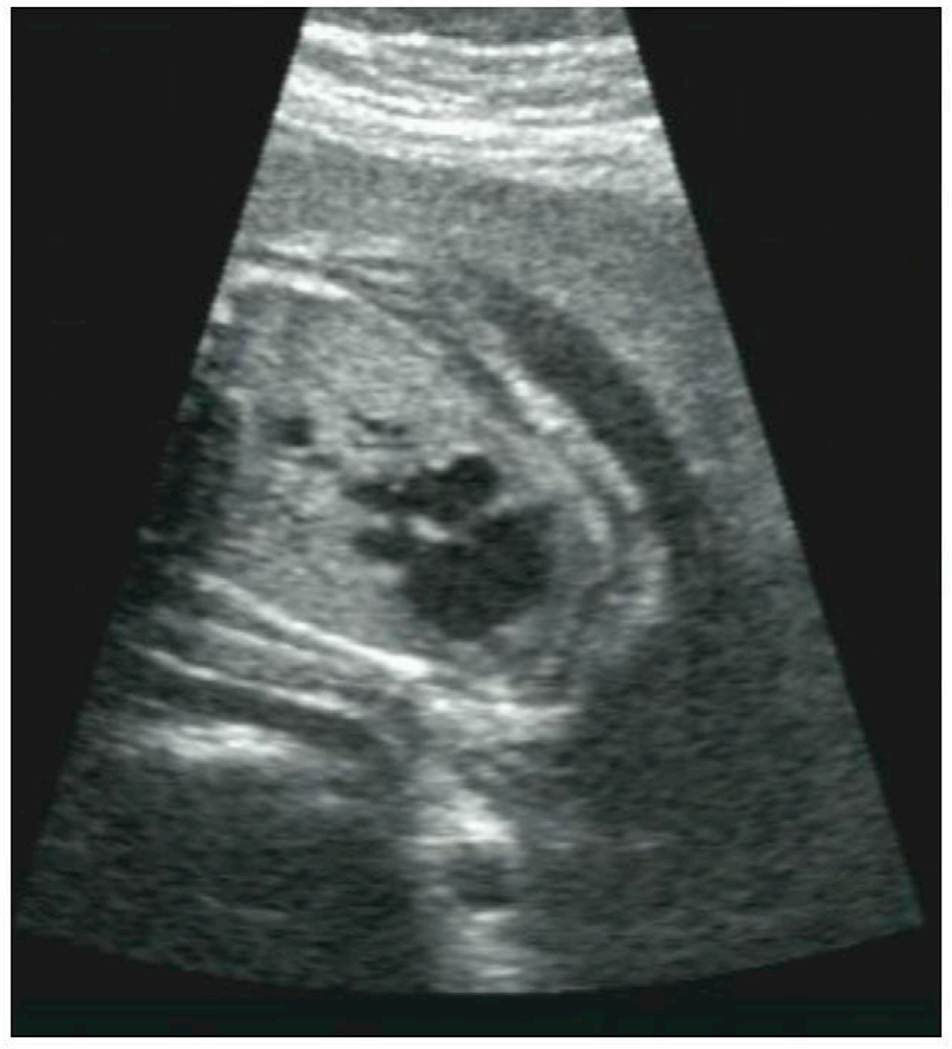
The fetus with congenital heart disease. Ultrasound scans of the fetus showed abnormal heart development, a single ventricle, pulmonary artery stenosis and situs inversus.

There was no early pregnancy reaction, no exposure to poison or radioactive substances, and no history of folic acid supplementation in early pregnancy. At 9 weeks of gestation, her mother came to our hospital for ultrasound examination and found the normal gestational sac and gestational age. At 15 weeks of gestation, her mother came to our hospital for routine prenatal examination and found the normal physical condition, low risk value of non-invasive prenatal testing (NIPT), and normal range of OGTT. However, at 27 weeks of gestation, the result of ultrasound examination showed fetal cardiac abnormalities include single ventricle, pulmonary artery stenosis and visceral inversion (Figure 1). More detailed tests were carried out 2 days later and confirmed that the proband had no ventricular septal structure. CDFI showed mild left atrioventricular regurgitation. Because of the continuous histories of adverse pregnancy outcomes, the parents hoped to clarify the genetic factors and guide the next birth.

### Molecular Analysis

**FIGURE 2:**
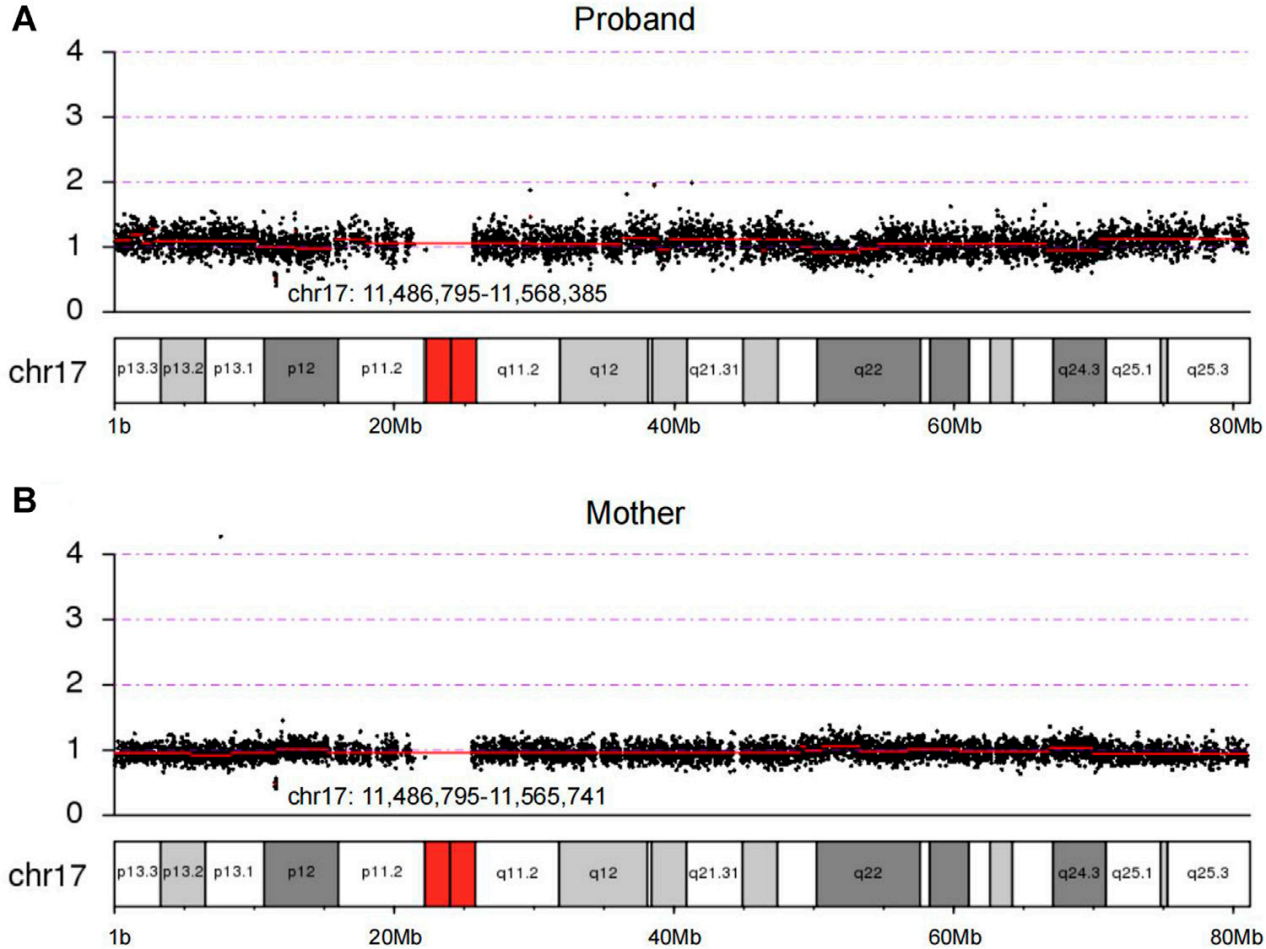
The loss of heterozygosity at 17p12 in the proband and the mother were detected by CNV-seq. **(A)** Copy number of chromosome 17 in the proband by sequencing. **(B)** Copy number of chromosome 17 in the proband’s mother by sequencing. The chr17:11,486,795–11,568,385 and chr17:11,486,795–11,565,741 showed in **(A)** and **(B)** were regions of deletion in the proband and her mother, respectively.

**FIGURE 3:**
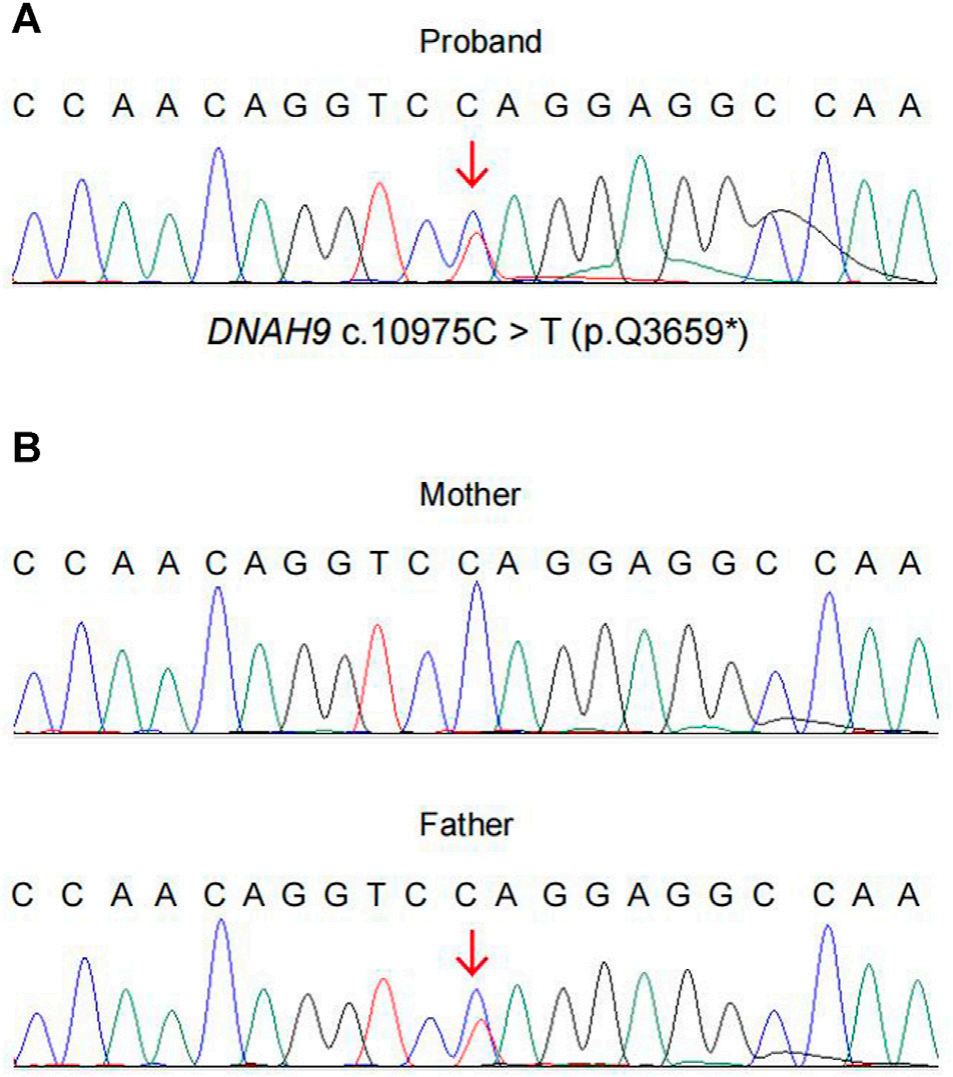
The evidence of the heterozygous nonsense mutation c.10975C> T (p.Q3659*) in the *DNAH9* gene. WES and sanger sequencing verified the heterozygous mutation *DNAH9* c.10975C> T in the proband **(A)** and her parents **(B)**.

Using CNV-seq, we identified an 81 kb deletion at chr17p12 (11,486,795–11,568,385) in the fetus (Figure 2A). This deletion region includes 1–15 exons of the *DNAH9* gene. After verified in her parents by CNV-seq, it is found that the mother had a 79 kb deletion at chr17p12 (11,486,795–11,565,741) (Figure 2B). The result implied the deletion at chr17p12 of the fetus was inherited from her mother. Moreover, we also performed the WES in the fetus and revealed another mutation at c.10975C>T (p.Q3659*) in the exon 57 of *DNAH9* gene (Figure 3A). Subsequently, sanger sequencing was used to verify this nonsense mutation in all family members and showed c.10975C>T *DNAH9* mutation was inherited from her father (Figure 3B). According to the American College of Medical Genetics and Genomics (ACMG) guidelines (Richards et al., 2015), both the 81 kb deletion at chr17p12 and c.10975C>T in *DNAH9* gene were predicted to be pathogenic mutations because of the evidence chain (PVS1+PM2+PP3).

### Effect of Mutations on Protein Structures

**FIGURE 4:**
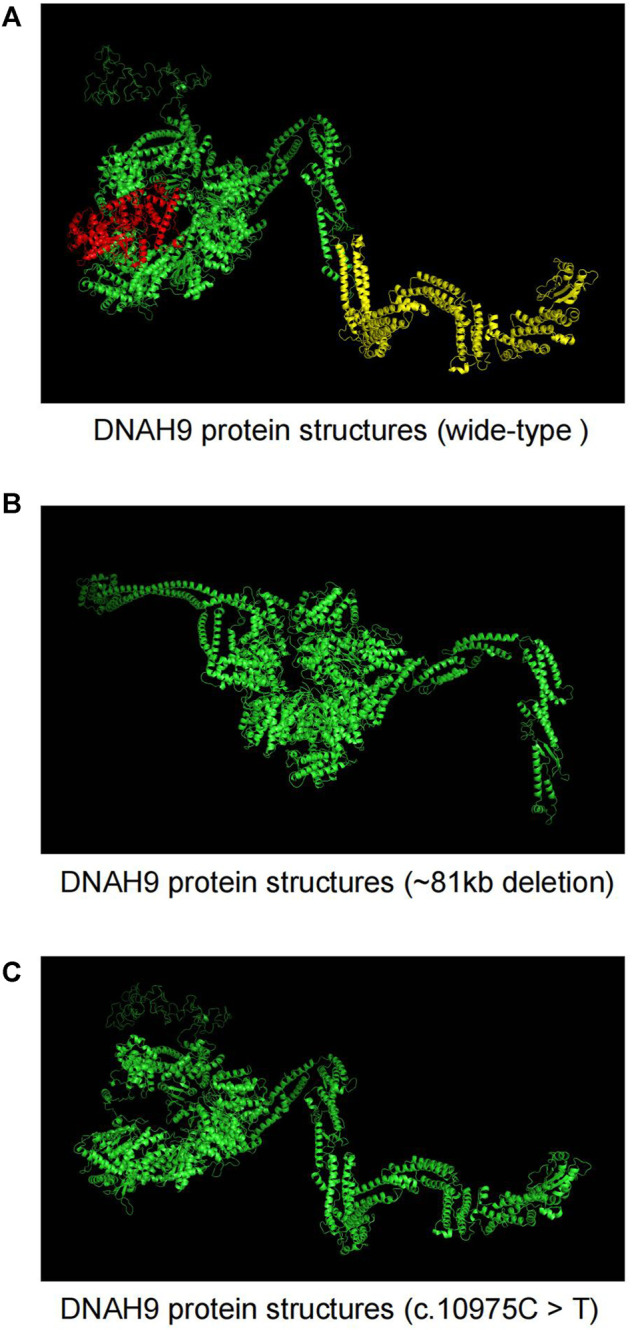
The Effects of *DNAH9* mutations on protein structure. **(A)** The wide-type structure of DNAH9 protein predicted by SWISS-MODEL. The yellow and red parts were modelled using exon 1–15 in *DNAH9* and amino acid residues from glutamine at position 3,659 to end. The mutant protein structures showed in **(B)** and **(C)** upon an 81 kb deletion at chr17p12 (11,486,795–11,568,385) or c.10975C> T mutation in *DNAH9*, respectively.

We used SWISS-MODEL web server (Waterhouse et al., 2018) to predict changes of DNAH9 protein structures when there were mutations in the sequence (Figure 4A). The deletion of 1–15 exons in *DNAH9* gene will alter the 3D structure on β-sheet (Figure 4B), while the c.10975C>T (p.Q3659*) mutation will change the 3D structure from α-helix to termination (Figure 4C). The combination of these structural changes would alter the conformation of the DNAH9 protein and affect the protein stability and binding facility.

## Discussion

Fetal CHD has become the most important birth defect type in China, accounting for the majority neonatal non-infectious diseases. However, the accuracy rate of prenatal diagnosis was only 36% by investigating 309 participants with CHD, including post-natal and fetal termination (Friedberg et al., 2009). The prenatal diagnosis rate of left ventricular outflow tract obstruction, transposition of great arteries and anomalous pulmonary venous drainage was the lowest. The traditional technology, ultrasound is the most effective non-invasive way of diagnosing CHD in fetal cases. However, because of the small meridian of the fetal heart, complex blood circulation, difficulties of capturing the fetal variable blood flow and the limitation of ultrasound itself, not all fetal CHD can be diagnosed by ultrasound in the prenatal setting. Although MRI can overcome shortcomings of the small field of vision and poor contrast of soft-tissue, the malformations of CHD patients are sometimes not limited to the heart. Additionally, many CHDs are accompanied by genetic changes, including chromosome aberration, single gene genetic defects, multi gene genetic defects and so on. Therefore, it is of great and urgent practical significance to make early and accurate prenatal genetic diagnosis of CHD in fetal cases by using next-generation sequencing technology.

Previous studies have proved that CMA is an effective tool to detect fetal genomic imbalances, including abnormalities of chromosome number and copy number variations (Xia et al., 2018). However, the price of CMA is relatively expensive. Copy number variation sequencing (CNV-seq), is a technology by using next-generation sequencing technology, can detect CNVs with high resolution in the whole genome. Compared with CMA, it can increase different sequencing depth to obtain more accurate information, and has the advantages of more flexible, fast, accurate and low operation cost (Duan et al., 2013). Moreover, CNV-seq technology has been applied to the genetic diagnosis of fetal CHD, recently (Zhu et al., 2016). In our study, we found that the proband had a loss of heterozygosity of 81 kb at 17p12 (11,486,795–11,568,385) by high-resolution CNV-seq. This deletion was within *DNAH9* gene. Then the parent’s samples were verified by CNV-seq, and found her mother was a carrier of this deletion.

The loss of heterozygosity in *DNAH9* alone should not cause CHD. To search for other potential genetic defects, WES and sanger sequencing of the proband and the parents were further performed and found one heterozygous nonsense mutation of *DNAH9* gene (c.10975C>T) in the proband inherited from her father. This mutation resulted in termination of translation at amino acid 3,659. With additional clinical evaluation, these two mutations meet ACMG criteria for classification as pathogenic of *DNAH9* mutations in this case.

In summary, through the combined application of high-through sequencing technologies (CNV-seq and WES), we established a probable cause of the couple’s poor pregnancy outcomes. The proband had compound heterozygous mutations, the deletion of 81 kb at 17p12 (11,486,795–11,568,385) and *DNAH9* c.10975C>T (p.Q3659*), inherited from each carrier parent. In addition, our results showed that the combination of CNV-seq and WES is an effective approach to prenatal diagnosis.

## Data Availability

The datasets for this article are not publicly available due to concerns regarding participant/patient anonymity. Requests to access the datasets should be directed to the corresponding authors.

## Abbreviations

ACMG, American College of Medical Genetics and Genomics; CHD, Congenital heart disease; CNV-seq, Copy number variation sequencing; *DNAH9*, *dynein axonemal heavy chain 9*; WES, Whole exome sequencing.
